# Supplementary material for: DFT study on CO2 capture using boron, nitrogen, and phosphorus-doped C20 in the presence of an electric field
Source: Sci Rep. 2024 May 29;14:12388. doi: 10.1038/s41598-024-62301-x (PMC11137125; doi:10.1038/s41598-024-62301-x)
Supplement: Supplementary file 1 — Supplementary Figures. [file 41598_2024_62301_MOESM1_ESM.pdf]

Supporting Information for: CO_2 capture using boron, nitrogen, and phosphorus-doped C_{20} in the present electric field: DFT study

Parham Rezaee,[*,†,‡] Shervin Alikhah Asl,[¶] Mohammad Hasan Javadi,[§] Shahab Rezaee,[‡] Razieh Morad,[†] Mahmood Akbari,[*,†] Seyed Shahriar Arab,[‖] and Malik Maaza[†]

†*UNESCO-UNISA-iTLABS Africa Chair in Nanoscience and Nanotechnology (U2ACN2), College of Graduate Studies, University of South Africa (UNISA), Pretoria, South Africa*

‡*Department of Biophysics, School of Biological Sciences, Tarbiat Modares University, Tehran, Iran*

¶*Unit 12, 3rd Floor, Sahel Apartment, Motahhari St., Ardabil, Iran*

§*Unit 1,NO. 17, Keyhan 2 Aly., Keyhan St., Ayatollah Kashani boulevard, Tehran, Iran*

‖*Department of Pediatrics, University of California, San Diego, La Jolla, CA 92093, USA*

E-mail: parham.rezaee@modares.ac.ir; makbari@tlabs.ac.za

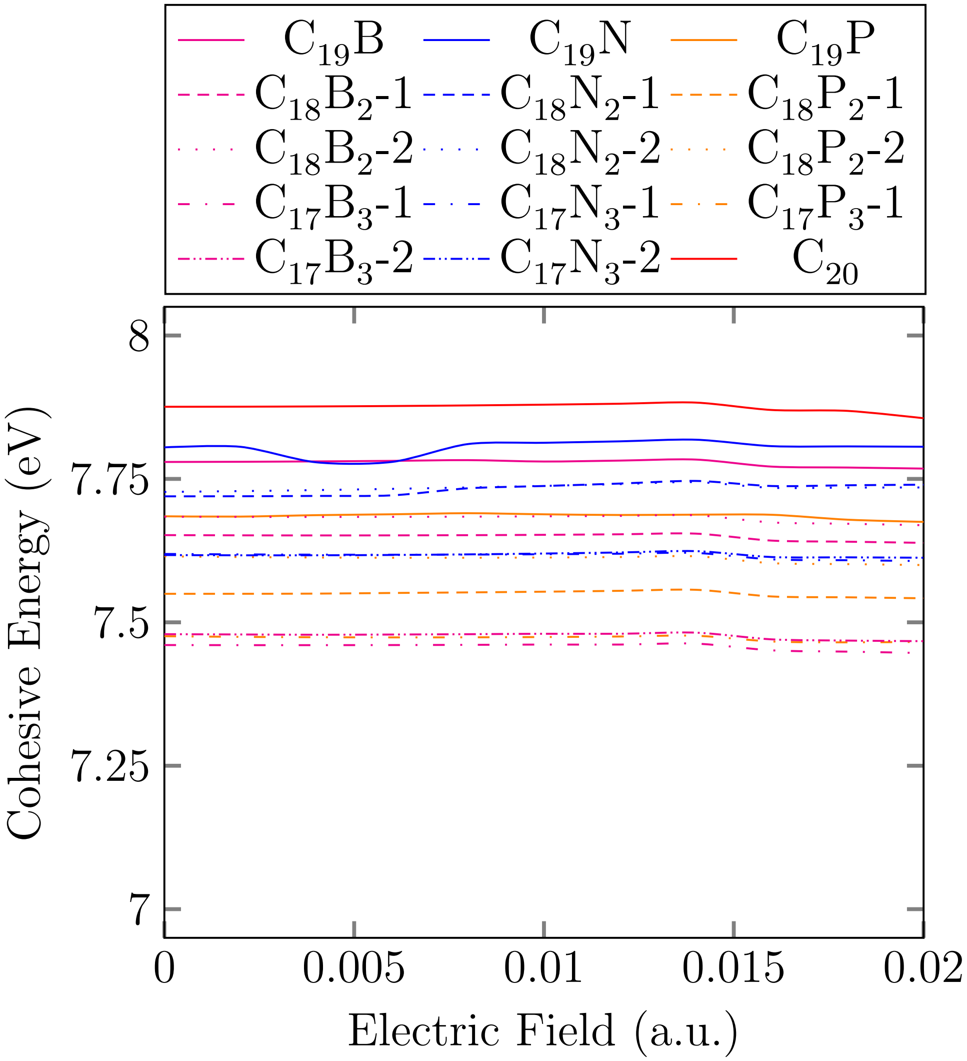

Figure S1: Variation of cohesive energy for C_{20} and $C_{20-n}X_n$ (n= 1, 2, and 3; X= B, N, and P) in 0–0.02 *a.u.* electric field.

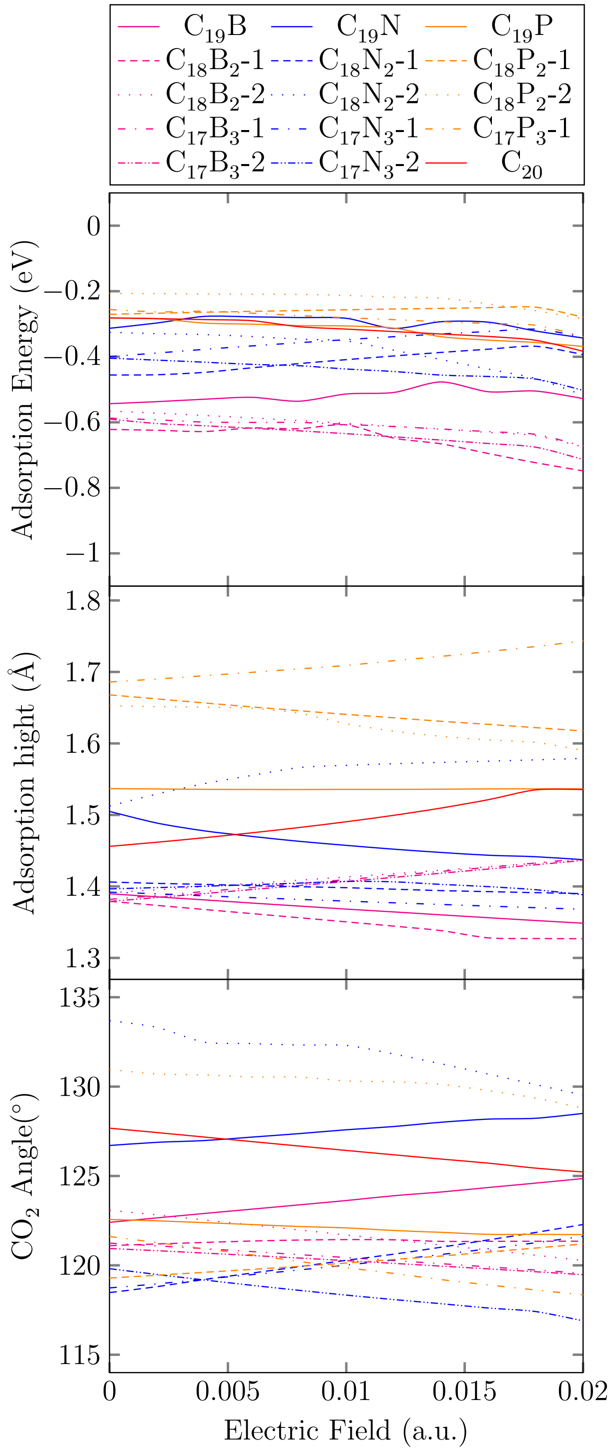

Figure S2: Variation of (a) adsorption energy (eV) (b) adsorption height (Å) (c) CO_2 angle (°) for C_{20} and $C_{20-n}X_n$ (n=1, 2, and 3; X=B, N, and P) in 0 – 0.02 *a.u.* electric field.
